# MicroRNA-423-5p Mediates Cocaine-Induced Smooth Muscle Cell Contraction by Targeting Cacna2d2

**DOI:** 10.3390/ijms24076584

**Published:** 2023-04-01

**Authors:** Derek M. Dykxhoorn, Huilan Wang, Andrea Da Fonseca Ferreira, Jianqin Wei, Chunming Dong

**Affiliations:** 1 Dr. John T. Macdonald Foundation Department of Human Genetics, University of Miami Miller School of Medicine, Miami, FL 33136, USA; 2 Department of Microbiology and Immunology, University of Miami Miller School of Medicine, Miami, FL 33136, USA; 3 John P. Hussman Institute for Human Genomics, University of Miami Miller School of Medicine, Miami, FL 33136, USA; 4 Interdisciplinary Stem Cell Institute, University of Miami Miller School of Medicine, Miami, FL 33136, USA; 5 Department of Medicine, University of Miami Miller School of Medicine, Miami, FL 33136, USA; 6 Section of Cardiology, Miami VA Health Systems, Miami, FL 33136, USA; 7 Biomedical Research Building, Suite 812, 1501 NW 10th Avenue, Miami, FL 33136, USA

**Keywords:** cocaine, intracellular calcium, microRNA, smooth muscle cells, blood pressure

## Abstract

Cocaine abuse increases the risk of atherosclerotic cardiovascular disease (CVD) and causes acute coronary syndromes (ACS) and hypertension (HTN). Significant research has explored the role of the sympathetic nervous system mediating the cocaine effects on the cardiovascular (CV) system. However, the response of the sympathetic nervous system alone is insufficient to completely account for the CV consequences seen in cocaine users. In this study, we examined the role of microRNAs (miRNAs) in mediating the effect of cocaine on the CV system. MiRNAs regulate many important biological processes and have been associated with both response to cocaine and CV disease development. Multiple miRNAs have altered expression in the CV system (CVS) upon cocaine exposure. To understand the molecular mechanisms underlying the cocaine response in the CV system, we studied the role of miRNA-423-5p and its target Cacna2d2 in the regulation of intracellular calcium concentration and SMC contractility, a critical factor in the modulation of blood pressure (BP). We used in vivo models to evaluate BP and aortic stiffness. In vitro, cocaine treatment decreased miR-423-5p expression and increased Cacna2d2 expression, which led to elevated intracellular calcium concentrations and increased SMC contractility. Overexpression of miR-423-5p, silencing of its target Cacna2d2, and treatment with a calcium channel blocker reversed the elevated SMC contractility caused by cocaine. In contrast, suppression of miR-423-5p increased the intracellular calcium concentration and SMC contractibility. In vivo, smooth muscle-specific overexpression of miR-423-5p ameliorated the increase in BP and aortic stiffness associated with cocaine use. Thus, miR-423-5p regulates SMC contraction by modulating Cacna2d2 expression increasing intracellular calcium concentrations. Modulation of the miR-423-5p—Cacna2d2—Calcium transport pathway may represent a novel therapeutic strategy to improve cocaine-induced HTN and aortic stiffness.

## 1. Introduction

Cocaine is a powerful sympathomimetic agent derived from the leaves of the *Erythroxylum coca* plant. With the exception of marijuana, cocaine remains the most commonly used drug of abuse and is the leading cause of drug abuse-related emergency room visits [1]. Cocaine is associated with a wide range of cardiovascular (CV) complications, including acute coronary syndromes, heart failure, cardiomyopathies, arrhythmias, stroke, hypertension (HTN), and aortic dissection [2,3,4,5,6,7,8]. Cocaine has multiple CV and prothrombotic effects that may contribute to the development of CV disease. Cocaine consumption causes a dose-dependent increase in blood pressure (BP) [9] and heart rate [10,11]. In addition, cocaine induces coronary and peripheral arterial vasoconstriction [12,13,14,15]. Cocaine stimulates the sympathetic nervous system by blocking the reuptake of catecholamines at the presynaptic adrenergic terminals, leading to the accumulation of catecholamines at the post-synaptic receptors and increasing the sensitivity of adrenergic nerve endings to norepinephrine (NE) [12,16]. Catecholamines are monoamines that are essential for maintaining homeostatic mechanisms in multiple tissues and organs. In the CVS, catecholamines regulate important biological functions, including heart rate and vascular tone. Calcium is an important mediator in catecholamine signaling with sustained exposure to catecholamines leading to Ca^2+^ overload in cardiomyocytes [17]. This increase in intracellular calcium concentrations ([Ca^2+^]i) can be mediated through the engagement of β-adrenergic receptors by catecholamines leading to a cascade of events that result in activation of cAMP-dependent protein kinase (PKA), phosphorylation of Ca^2+^ channels and Ca^2+^ influx [18,19]. Calcium overload [20] is just one mechanism that was proposed to be responsible for the cocaine-associated CV complications with additional mechanisms, including oxidative stress [21,22] and mitochondrial dysfunction [17,23]. However, all of these processes were studied in the context of catecholamines. Emerging evidence suggests that, although catecholamines play important roles, additional mechanisms may be mediating cocaine-induced CV complications. For example, when cocaine or cocaine methiodide (CM, which does not enter the CNS) was administered 5 min before administering a calcium channel blocker Nifedipine, neither cocaine nor CM changed the effect of NE on heart rate and blood pressure (BP) [24]. This observation suggests that the NE potentiation mechanism cannot fully explain cocaine’s effects on the CV system.

To better understand the molecular mechanisms underlying the effects of cocaine on the CV system and to identify new pathways that may mediate these effects, we recently performed small RNAseq and RNASeq on aortas derived from mice treated with cocaine, CM, or saline (control) [25]. Micro (mi) RNAs are small non-coding RNAs that post-transcriptionally regulate gene expression by binding to the 3′ UTR of target transcripts leading to translational repression and/or mRNA degradation [26,27,28]. MiRNAs have been shown to play important roles in CV development and disease, vascular aging, and response to drug exposure [29,30,31,32,33]. The role of miRNAs in mediating the effects of cocaine exposure on the central nervous system has been extensively examined. For example, miR-212 was shown to play a prominent role in the vulnerability to cocaine addiction by controlling two complementary mechanisms—amplification of CREB signaling [34] and reduction in MeCP2/BDNF transmission in the striatum [35]. Additional miRNAs were found to be dysregulated in various regions of the brain following cocaine exposure (reviewed in [36]). However, little is known about the role of miRNA pathways in mediating the cocaine effects in the CV system. Therefore, our RNAseq data sets were used to identify miRNAs and mRNAs that were differentially expressed in the cocaine/CM compared to saline treatment and whose expression were inversely correlated. We previously reported that miR-30c-5p was upregulated in the aortas of mice treated with cocaine. MiR-30c-5p directly targeted the redox molecule malic enzyme 1 (Me1), leading to increased reactive oxygen species (ROS) levels. Interestingly, the silencing of miR-30c-5p in mouse aortas abrogated the cocaine-induced increase in ROS, resulting in partial normalization of BP and aortic stiffness [33]. In this current study, we investigated the potential involvement of an additional miRNA—mRNA pathway in mediating cocaine effects in the CV system implicated by our previous RNAseq analysis [25]. Specifically, we showed that miR-423-5p was downregulated in cocaine-exposed compared to the control-treated mouse aortas. To identify the putative target of miR-423-5p, we curated a list of mRNAs whose expression was inversely correlated with that of miR-423-5p. From this list of inversely correlated mRNAs, in silico miRNA target predictive tools were used to identify putative targets of miR-423-5p. This analysis identified *Cacna2d2*—the gene encoding the α2δ-2 subunit of voltage-dependent calcium channels—as a putative target of miR-423-5p. Luciferase reporter assays confirmed that *Cacna2d2* was a direct target of miR-423-5p. The silencing of Cacna2d2—either through the overexpression of miR-423-5p or a Cacna2d2 siRNA—led to decreased contractility of smooth muscle cells (SMCs) in vitro. Importantly, in vivo overexpression of miR-423-5p in mouse SMCs reduced cocaine-induced elevation of BP and aortic stiffness. These results support an important role for the miR-423-5p—Cacna2d2 axis, in addition to our previously characterized miR-30c-5p—ME1—ROS pathway, in the regulation of vasoconstriction resulting from cocaine exposure.

## 2. Results

### 2.1. Cocaine and Cocaine Methiodide (CM) Exposure Increased BP, Aortic Stiffness, and Alters miR-423-5p and Cacna2d2 Expression in the Mouse Aorta

**Figure 1:**
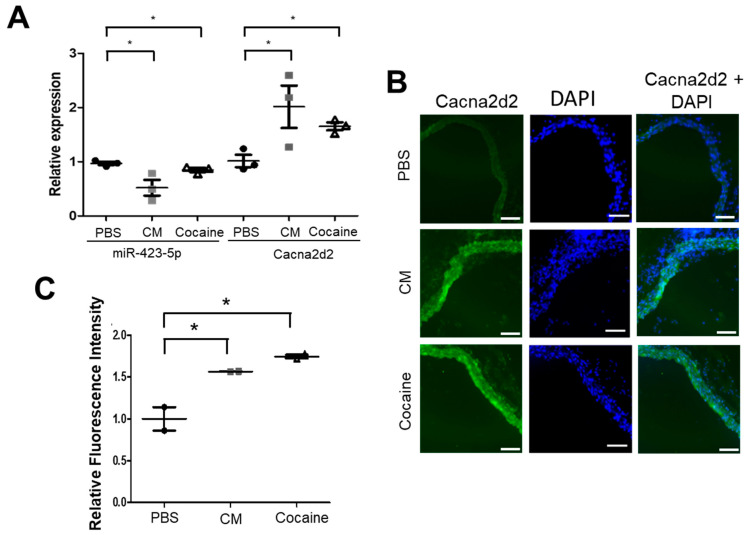
Cocaine and CM suppress miR-423-5P expression and increase Cacna2d2 expression in the mouse aortas. (**A**) QRT-PCR analysis was performed using RNA extracted from aortas isolated from mice treated with saline, cocaine, or CM for 10 consecutive days. Cocaine and CM resulted in decreased miR-423-5p expression and increased Cacna2d2 expression compared to saline treatment. Black circles = saline treatment, gray squares = CM treatment, open triangle = cocaine treatment (* *p* < 0.05 vs. saline). (**B**) Immunohistochemical (IHC) analysis of Cacna2d2 levels in aortic sections showed cocaine and CM treatment induced increased levels of Cacna2d2 protein expression compared to saline. Scale bars = 100 µm, magnification 20× (PBS group), 50 µm magnification 40× (CM and Cocaine groups) (**C**) The intensity of Cacna2d2 positive staining was quantified using Image J software. Student’s two tailed *t*-test was used for all statistical analysis (* *p* < 0.05 vs. saline).

**Figure 2:**
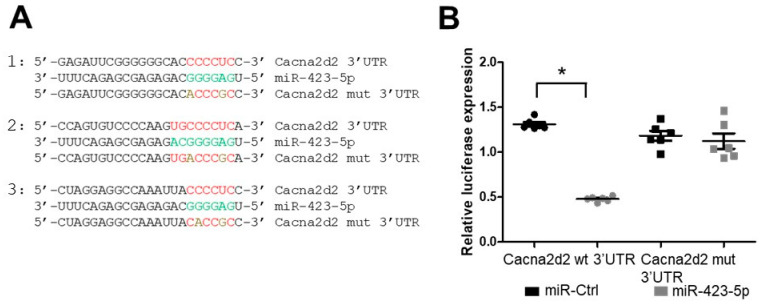
miR-423 has putative binding sites in Cacna2d2. (**A**) In silico analysis identified 3 putative miR-423-5p binding sites in the Cacna2d2 3′ UTR (site 1: 426–448; site 2: 812–834; site 3: 1098–1121 based on the NM_020263 transcript variant). Point mutations were introduced in these three sites to determine the potential binding of miR-423-5p to the Cacna2d2 3′UTR. Green lettering represents the seed sequence of miR-423-5p while red lettering represents the seed match of the miR-423-5p binding site on the Cacna2d2 mRNA. (**B**) Luciferase reporter assays were performed by co-transfection of HEK-293 cells with luciferase reporter constructs containing the WT or mutant Cacna2d2 3′ UTR and either the miR-423-5p or a non-specific control miRNA (miR-Ctrl) expression vector. Gaussia luciferase activities (GLuc), measured and normalized to secreted alkaline phosphatase (SeAP) activities, show that Cacna2d2 is a direct target of miR-423-5p. Student’s two tailed *t*-test was used for all statistical analysis (* *p* < 0.05 vs. miR-Ctrl). Black circle = Cacna2d2 wt 3′ UTR + miR-Ctrl, gray circles = Cacna2d2 wt 3′ UTR + miR-423-5p, black squares = Cacna2d2 mutant 3′ UTR + miR-Ctrl, gray squares = Cacna2d2 mutant 3′ UTR + miR-423-5p.

Cocaine nas been linked to increased risk for CV disease [9,37,38,39]. To assess the impact that cocaine exposure has on miRNA expression in the aorta, we developed a model of cocaine use/abuse in C57BL/6 mice [33]. In this model, 8–10 week old mice were given daily intraperitoneal injections of cocaine, CM, or saline. Compared to the saline-treated mice, both systolic and diastolic BP were significantly increased in the cocaine and CM-treated animals throughout the treatment course, as previously described [33]. In addition, the cocaine- and CM-treated mice showed increased aortic stiffness, as measured by pulse wave velocity (PWV) on day 0 and then, two days following the final injection of cocaine [33]. RNAseq and small RNAseq analysis were performed on aortas harvested from these treated mice. Differential gene expression analysis was performed to identify miRNAs and mRNAs that were differentially expressed between the cocaine/CM-treated and the control-treated mice [25]. We showed that cocaine exposure resulted in an increase in miR-30c-5p levels leading to decreased expression of Malic enzyme-1 (Me-1) and increased reactive oxygen species. Reducing miR-30c-5p expression or treating mice with the free oxygen radical scavenger with N-acetyl cysteine (NAC) led to reduced pulse wave velocity—a measure of aortic stiffness [33]. Consistent with our small RNAseq data, quantitative real-time PCR (qRT-PCR) analysis showed that miR-423-5p expression decreased in the aortas of cocaine- and CM-treated mice compared to the control (saline) treatment (Figure 1A). In silico analysis of miRNA targets using prediction programs—TargetScan and miRdb—identified Cacna2d2 as a putative miR-423-5p target [40,41]. Furthermore, the expression of Cacna2d2 was elevated in cocaine- and CM-treated compared to the control (saline)-treated mouse aortas (Figure 1A). Immunohistochemical staining of the mouse aortas confirmed the increase in Cacna2d2 expression in response to CM and cocaine treatment, relative to saline control (Figure 1B,C). To determine if the anticorrelation of miR-423-5p with Cacna2d2 expression was due to direct inhibition of Cacna2d2 by miR-423-5p, luciferase reporter assays were performed. The 3′UTR of Cacna2d2 was cloned into a luciferase reporter construct which was transfected into HEK293T cells along with either miR-423-5p or a non-targeting, control miRNA (miR-Ctrl). The co-transfection of miR-423-5p with the Cacna2d2 3′UTR construct showed a significant decrease in luciferase activity compared to miR-Ctrl treatment (Figure 2A,B). Three potential miR-423-5p binding sites—one highly conserved site and two with lower binding properties—were identified in silico in the 3′ UTR of Cacna2d2 (site 1: 426–448; site 2: 812–834; site 3: 1098–1121 based on the NM_020263 transcript variant) (Figure 2A). Site-directed mutagenesis was used to introduce mutations that should disrupt the interaction between miR-423-5p and the three potential miR-423-5p binding sites in the Cacna2d2 3′ UTR. Similar to the miR-Ctrl treatment, the co-transfection of miR-423-5p with the mutated version of Cacna2d2 3′ UTR (mtCacna2d2 3′ UTR) was unable to silence luciferase expression (Figure 2B)**.** These results showed that Cacna2d2 is a direct target of miR-423-5p.

### 2.2. Cocaine and CM Treatment Increased Intracellular Free Calcium [Ca^2+^] and Induce Primary Mouse Aortic SMCs Contraction

**Figure 3:**
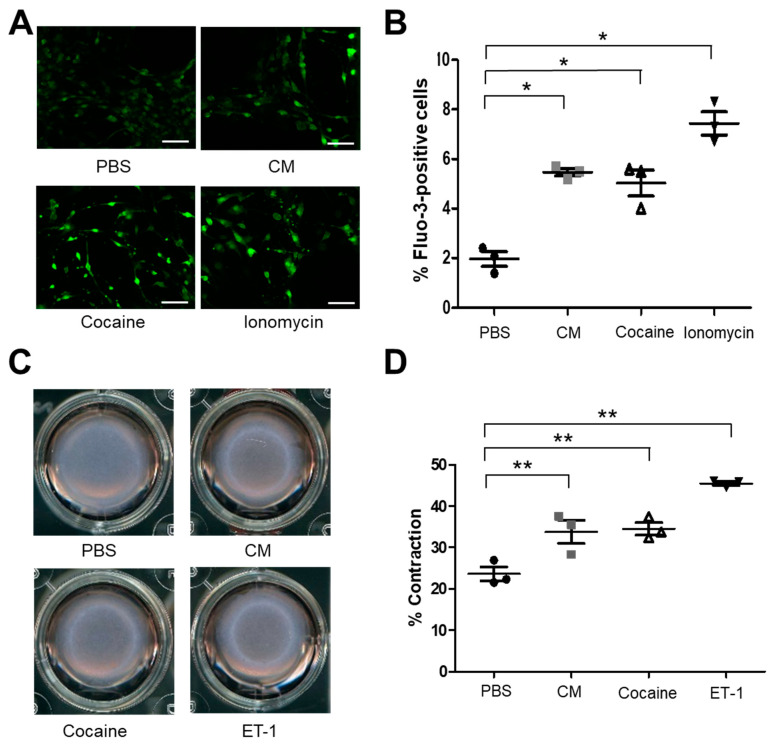
Cocaine and CM increase intracellular free calcium [Ca^2+^] and induce primary mouse aortic SMC contraction. (**A**) Fluorescence image analysis of free cytosolic Ca^2+^ in mouse aortic SMCs treated with PBS, CM, cocaine, or the calcium ionophore ionomycin (positive control) and stained with the calcium specific dye Fluo-3 AM show that cocaine and CM increase the level of intracellular free calcium in SMCs. Scale bars = 400 μm. (**B**) Quantification by Image J software analysis fluorescence positive cells. Black circles = saline treatment, gray squares = CM treatment, open triangles = cocaine treatment, closed triangles = Ionomycin treatment (* *p* < 0.05 vs. saline). (**C**) Mouse Aortic SMCs were exposed to saline, cocaine, CM or the vasoactive peptide ET-1 (positive control), and their contractility was measured using the collagen gel contraction assay. Representative images of SMC contractility in the collagen contraction assay show increased SMC contraction in response to cocaine and CM. (**D**) Quantification of collagen gel contraction show both cocaine and CM substantially increase SMC contraction relative to PBS Student’s two tailed *t*-test was used for all statistical analysis (** *p* < 0.05 vs. PBS). Black circles = saline treatment, gray squares = CM treatment, open triangles = cocaine treatment, closed triangles = Endothelin-1 treatment.

The mice in the cocaine abuse/use model showed elevated BP and aortic stiffness [33]. Cocaine abuse was previously shown to induce vasoconstriction [42,43,44,45,46]. Ca^2+^ plays a central role in excitation-contraction coupling in vascular SMCs among other functions (reviewed in [47]). Therefore, the regulation of intracellular Ca^2+^ concentration ([Ca^2+^]i) is crucial for the proper functioning of SMCs. Cacna2d2 encodes a subunit of the L-type Cav1.2 channels that are key regulators of Ca^2+^ influx and myogenic tone [48,49,50]. To examine whether cocaine treatment caused the [Ca^2+^]i changes in primary mouse aortic SMCs, intracellular Ca^2+^ levels were measured in mouse SMCs in the presence or absence of cocaine or CM. SMCs were cultured and treated with saline, cocaine, CM, or the calcium ionophore ionomycin, and the cells were stained with the Ca^2+^-specific fluorescent dye, 3-AM (Figure 3A,B). Fluorescence imaging of cocaine or CM treated cells showed an increase in intracellular Ca^2+^ compared to the saline control treated cells (Figure 3A). Flow cytometric analysis of Fluo-3 AM stained cells confirmed the increase in intracellular Ca^2+^ following treatment with cocaine or CM (Figure 3B). The level of fluorescence seen in response to cocaine or CM treatment was similar to the level observed when the cells were treated with the ionophore ionomycin (positive control). These results show that treatment with cocaine and CM led to an increase in [Ca^2+^]i levels in SMCs.

To test the effect of cocaine and CM on the contractility of SMCs, a cell contraction assay was performed by embedding the SMCs in a collagen matrix that was attached to the well of the tissue culture plate. The initial period of attachment to the tissue culture plate allows for mechanical loading and stress fiber formation. After a total of 48 h following plating, the collagen matrices were gently released from the tissue culture plate resulting in mechanical unloading and contraction of the cell-embedded collagen matrix and the contraction index measured. Treatment with cocaine or CM resulted in increased contraction of the collagen matrix compared to the PBS-treated cells (Figure 3C,D). Figure 3C shows bright field images of the matrix embedded with SMCs and treated with saline, cocaine, CM, and endothelin-1 (potent endogenous vasoconstrictor molecule). The contractile index was quantified for each treatment (Figure 3D). Treatment with cocaine or CM increased SMC contraction compared to the saline treatment. This increase in contractility was similar to that observed with endothelin-1 treatment (positive control for SMC contraction). These results show that cocaine and CM increased the [Ca^2+^]i and contractility of SMCs compared to PBS-treated cells.

### 2.3. Modulation of the miR-423—Cacna2d2 Axis Altered Intracellular Free Calcium [Ca^2+^]i and Contractility of Mouse Aortic SMCs

**Figure 4:**
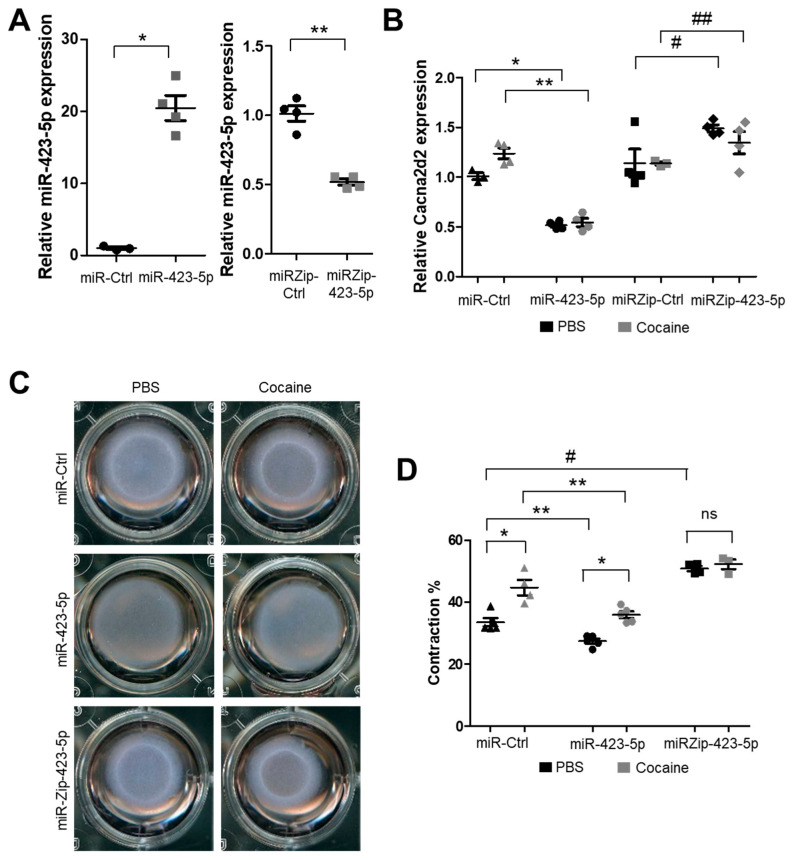
The miR-423-5p-Cacna2d2 axis mediates cocaine-induced SMC contraction. SMCs were transduced with lentiviral vectors encoding control miRNA (miR-Ctr), miR-423-5p, Control miRNA antagonist (miRZip-Ctr) or the miR-423-5p antagonist (miRZip-423-5p). (**A**) qRT-PCR analysis shows that miR-423-5p expression was increased in the miR-423-5p-transduced cells and decreased in miR-Zip-423-5p-transduced cells. Left panel, black circles = miR-Ctrl treatment, gray squares = miR-423-5p treatment; Right panel, black dots = miRZip-Ctrl treatment, gray squares = miRZip-423-5p treatment. (* *p* < 0.05 vs. miR-Ctrl; ** *p* < 0.05 vs. miRZip-Ctrl). (**B**) MiR-423-5p overexpression led to a significant decrease in Cacna2d2 expression both in the absence (PBS) or presence of cocaine (*p* < 0.05 compared to miR-Ctr). Conversely, silencing of miR-423-5p by miRZip-423-5p treatment led to increased Cacna2d2 expression in the presence or absence of cocaine. Black triangles = miR-Ctrl + saline, gray triangles = miR-Ctrl + cocaine, black circles = miR-423-5p + saline, gray circles = miR-423-5p + cocaine, black squares = miRZip-Ctrl + saline, gray squares = miRZip-Ctrl + cocaine, black diamonds = miRZip-423-5p + saline, gray diamonds = miRZip-423-5p + cocaine (* *p* < 0.05 vs. miR-Ctrl + saline; ** *p* < 0.05 vs. miR-Ctrl + cocaine; # *p* < 0.05 vs. miRZip-Ctrl + saline; ## *p* < 0.05 vs. miRZip-Ctrl + Cocaine). (**C**) Aortic SMCs were treated with lentiviral vectors encoding miR-423-5p, miR-Ctrl, miRZip-423-5p or miRZip-Ctrl, and the transduced SMCs were seeded on collagen gel with or without treatment of cocaine. Representative images of the collagen contractility assay show miR-423-5p abrogates, whereas miRZip-423-5p potentiates cocaine-induced SMC contraction. (**D**) Quantification of collagen gel contractility assays confirm that cocaine-induced SMC contraction is mediated, at least in part, by miR-423-5p. Student’s two tailed *t*-test was used for all statistical analysis (ns: not significant; * *p* < 0.05 vs. saline; ** *p* < 0.05 vs. miR-Ctrl; # *p* < 0.05 vs. miR-Ctrl + saline). Black triangles = miR-Ctrl + saline, gray triangles = miR-Ctrl + cocaine, black circles = miR-423-5p + saline, gray circles = miR-423-5p + cocaine, black squares = miRZip-523-5p + saline, gray squares = miRZip-423-5p = cocaine.

Treatment with cocaine or CM increased [Ca^2+^]i levels and the contractility of SMCs. We sought to determine if cocaine/CM were exerting their effect on SMC contractility through the miR-423-5p—Cacna2d2 pathway. To that end, the levels of miR-423-5p and Cacna2d2 were modulated in SMCs, and these cells were assayed for the impact of these changes on SMC contractility. As expected, overexpression of miR-423-5p in SMCs led to a significant decrease in Cacna2d2 expression (Figure 4A,B). This decrease in Cacna2d2 expression was seen even in cells that were exposed to cocaine (Figure 4B). Conversely, the silencing of miR-423-5p using the miR-423-5p antagomir, miR-Zip-423-5p, led to an increase in Cacna2d2 levels both in the absence and in the presence of cocaine (Figure 4B).

To determine if the increased SMC contraction in response to cocaine and CM was mediated via the miR-423-5p—Cacna2d2 pathway, collagen gel contraction assays were performed in SMCs transduced with lentivirus encoding miR-423-5p or the miR-423-5p antagomir—miRZip-423-5p. Overexpression of miR-423-5p blocked cocaine-induced SMC contraction compared to the control miRNA (miR-Ctrl) treated cells. Interestingly, miR-423-5p overexpression also led to a decrease in contractility in the absence of cocaine treatment (Figure 4C,D). By contrast, silencing of miR-423-5p expression (miR-Zip-423-5p-transduced SMCs) showed increased SMC contraction, irrespective of cocaine treatment, compared to miR-Ctrl-transduced SMCs. These results show that the modulation of the cocaine responsive miRNA—miR-423-5p—altered SMC contractility with the overexpression of miR-423-5p suppressing SMC contraction while, conversely, the silencing of miR-423-5p (miRZip-423-5p treatment) promoted SMC contraction.

**Figure 5:**
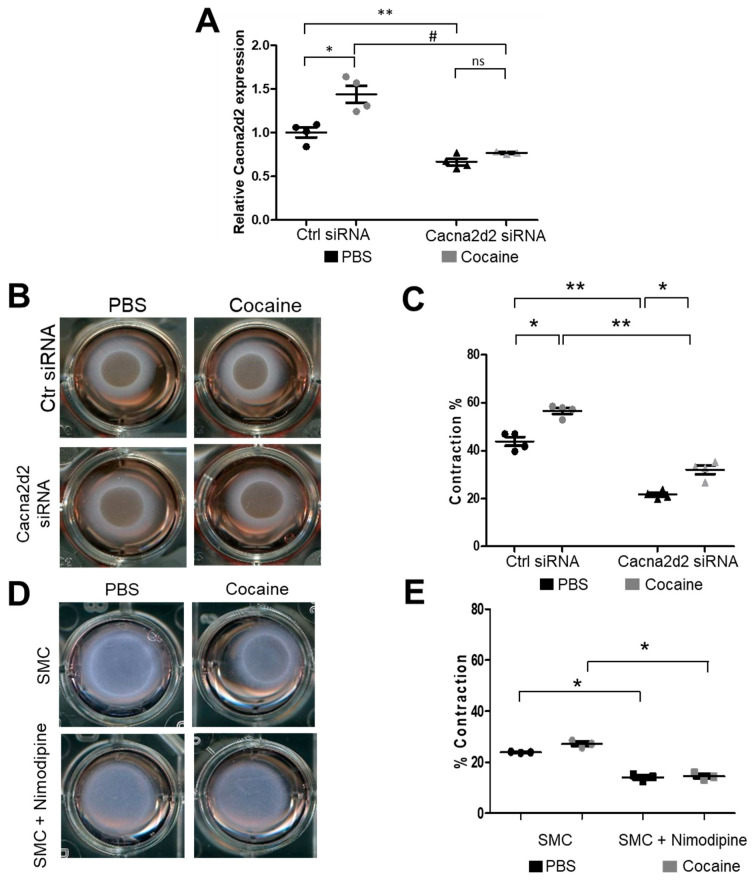
Cacna2d2 and intracellular calcium concentration mediate cocaine-induced SMC contraction. (**A**) Cacna2d2 siRNA decreased Cacna2d2 mRNA expression in SMCs compared with Ctr siRNA in the absence or presence of cocaine. Black circle = Ctrl siRNA + saline, gray circle = Ctrl siRNA + cocaine, black triangle = Cacna2d2 siRNA + saline, gray triangle = Cacna2d2 siRNA + cocaine (ns: not significant; * *p* < 0.01 vs. Ctrl siRNA + saline; ** *p* < 0.01 vs. Ctrl siRNA + saline; # *p* < 0.01 vs. Ctrl siRNA + cocaine). (**B**) Representative images of the collagen contractility assay in SMCs in which Cacna2d2 was silenced in the absence or presence of cocaine show that the silencing of Cacna2d2 in SMCs leads to a decrease in SMC contractility compared to Ctr siRNA. (**C**) The effects of Cacna2d2 silencing on SMC contractility was measured by image J analysis of the collagen gel contractility assay. Black circle = Ctrl siRNA + saline, gray circle = Ctrl siRNA + cocaine, black triangle = Cacna2d2 siRNA + saline, gray triangle = Cacna2d2 siRNA + cocaine (* *p* < 0.05 vs. saline; ** *p* < 0.01 vs. Ctrl siRNA). (**D**) Treatment of SMCs with the L-type calcium channel antagonist Nimodipine (NM) reduces SMC contractility (compared to saline treatment) and abrogates the effect of cocaine on SMC contractility. (**E**) Quantification of collagen gel contraction images show NM inhibited SMC contraction compared with PBS treatment and abrogated SMC contraction induced by cocaine. Student’s two tailed *t*-test was used for all statistical analysis (* *p* < 0.05 vs. Ctrl siRNA). Black circles = SMC + saline, gray circles = SMC + cocaine, black squares = SMC + Nimodipine + saline, gray squares = SMC + Nimodipine + cocaine.

Each miRNA has the potential to target multiple mRNAs [24]. Therefore, we sought to confirm that the effect of miR-423-5p on SMC contraction was regulated through Cacna2d2. To that end, Cacna2d2 expression was silenced in SMCs (Cacna2d2 siRNA) both in the presence and absence of cocaine (Figure 5A). Cacna2d2 silencing significantly abrogated the contractility of the SMCs in the collagen matrix contractility assay upon cocaine exposure, compared to the control siRNA-treated cells (Figure 5B,C). To further confirm the role of [Ca^2+^]i in cocaine-induced SMC contraction, SMCs were treated with the L-type voltage gated Ca^2+^ channel blocker Nimodipine (NM) in the presence or absence of cocaine. Treatment with NM resulted in decreased [Ca^2+^]i (Figure S1) compared to control treatment. Importantly, NM blocked the cocaine-induced SMC contraction (Figure 5D,E), showing that the SMC contraction was mediated through altered intracellular Ca^2+^ levels. Remarkably, the effects of NM on cocaine-induced [Ca^2+^]i and SMC contraction were similar to that seen with Cacna2d2 silencing– either mediated by the overexpression of miR-423-5p (Figure 4C,D) or the siRNA-mediated silencing of Cacna2d2 (Figure 5B,C). These data confirm that cocaine-induced SMC contraction was mediated via the miR-423—Cacna2d2—[Ca^2+^]i axis.

### 2.4. miR-423-5p Ameliorated Cocaine-Induced Increases in BP and Aortic Stiffness In Vivo

**Figure 6:**
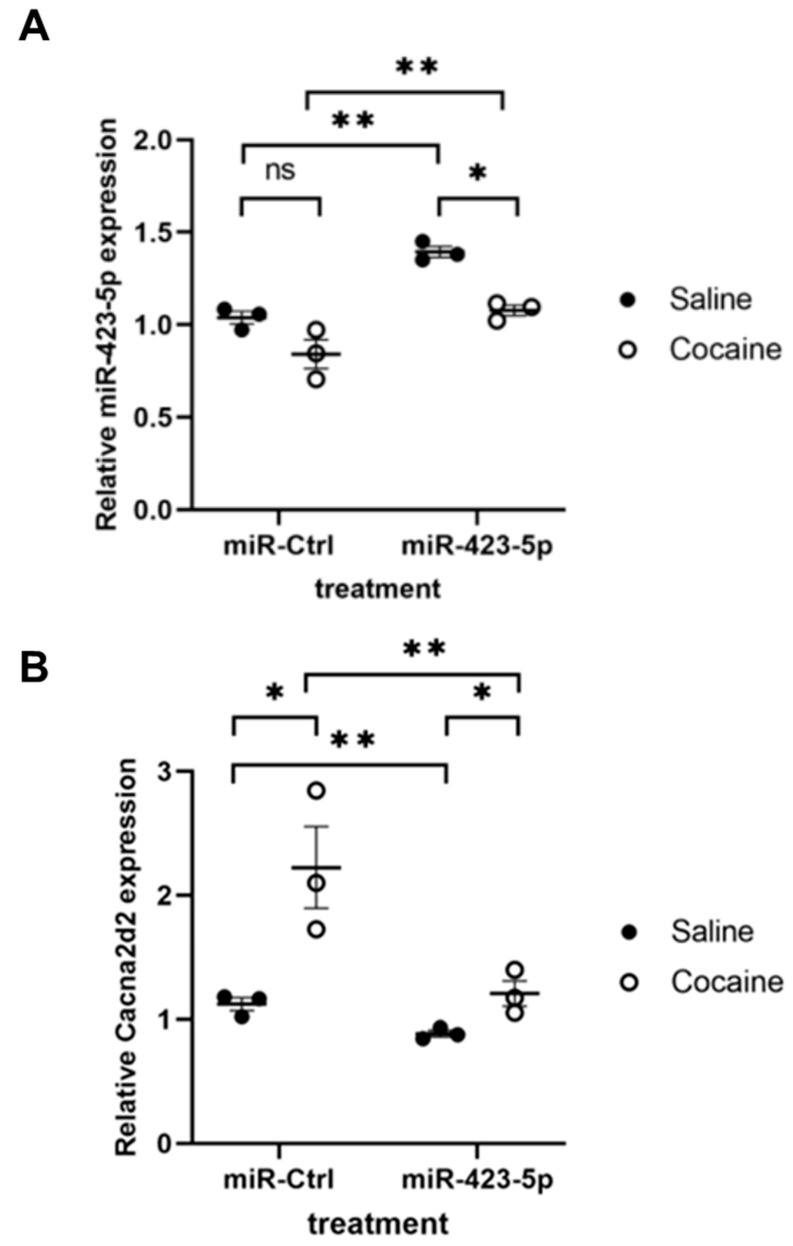
Treatment with miR-423-5p vector leads to increased expression of miR-423 and decreased expression of Cacna2d2 in mouse aortas. (**A**) Quantitative real-time PCR (qRT-PCR) analysis shows that the systemic treatment of mice with lentivirus expressing tmiR-423-5p from the smooth muscle- specific promoter Sm22a leads to an elevation of miR-423-5p compared to the control mice (vector control treatment) when measured in the aortas harvested from the cocaine (open circles) and saline (closed circles) treated mice. (ns: not significant; * *p* < 0.05 vs. miR-Ctrl; ** *p* < 0.05 vs. Saline). (**B**) qRT-PCR analysis also shows that miR-423-5p overexpression decreases Cacna2d2 expression in aortas compared with vector control treated mice in the absence or presence of cocaine. Student’s two tailed *t*-test was used for all statistical analysis (* *p* < 0.05 vs. saline; ** *p* < 0.05 vs. miR-Ctrl).

**Figure 7:**
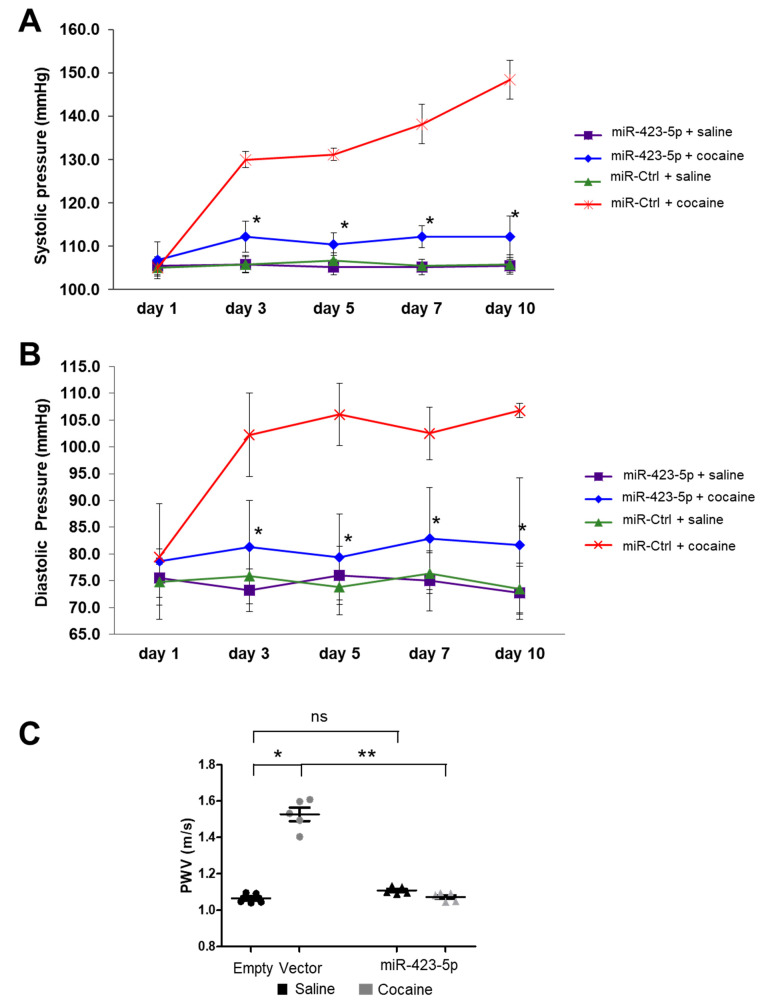
SMC-specific miR-423-5p ameliorated cocaine-induced increase BP and aortic stiffness in mice. Mice were injected with lentivirus encoding miR-423-5p from the smooth muscle-specific promoter (Sm22) or vector control before exposure to cocaine or PBS for 10 consecutive days. Pretreatment with miR-423-5p resulted in a partially reduction in the cocaine-induced (**A**) systolic and (**B**) Diastolic BP elevation. (* *p* < 0.05 vs. miR-Ctrl + cocaine). (**C**) Aortic stiffness was measured by pulse wave velocity (PWV) two days following the final treatment with cocaine. Pretreatment of the mice with the miR-423-5p expressing vector abrogates cocaine-induced elevation in aortic stiffness. Student’s two tailed *t*-test was used for all statistical analysis (ns: not significant; * *p* < 0.05 vs. miR-Ctrl; ** *p* < 0.01 vs. miR-Ctrl + cocaine). Black circles = empty vector + saline, gray circles = empty vector + cocaine, black triangle = miR-423-5p + saline, gray triangles = miR-423-5p + cocaine.

In primary SMCs, cocaine exposure resulted in an elevated [Ca^2+^]i and induced SMCs contraction by modulating the miR-423-5p—Cacna2d2 pathway. To determine if the miR-423-5p—Cacna2d2 axis contributed to the elevated BP and aortic stiffness seen in the mouse model of cocaine use/abuse, mice were injected with lentivirus that overexpress miR-423-5p under the control of a SMC-specific *Sm22*α promoter or an empty vector control lentivirus (Figure 6A). These mice were then exposed to daily intraperitoneal injection of cocaine over the course of 10 days. Aortas harvested from control-transduced and cocaine-treated mice showed increased Cacna2d2 expression compared to the saline treatment. MiR-423-5p lentivirus transduction reduced Cacna2d2 expression in both cocaine and saline treated mice aortas compared to the control lentiviral transduction (Figure 6B). BP was measured throughout the course of the experiment with both systolic and diastolic BP showing a progressive increase over the course of the experiment in the control-transduced mice following cocaine treatment compared to the saline treatment (Figure 7A,B). However, pre-treatment with the miR-423-5p overexpression vector partially abrogated the cocaine-induced increase in both systolic and diastolic BP compared to the control treatment (Figure 7A,B). Aortic stiffness was measured at day 0 and then, 2 days following the last cocaine treatment by pulse wave velocity (PWV). Similar to the results of the BP analysis, control-transduced mice showed increased aortic stiffness after cocaine exposure when compared with saline treatment. Importantly, transduction of the mice with miR-423-5p expressed from the Sm22α promoter was able to ameliorate the increase in PWV seen with cocaine treatment (Figure 7C). Collectively, these results indicate that the cocaine-induced increases in BP and aortic stiffness can be, at least partially, abrogated by miR-423-5p overexpression, providing in vivo evidence that the miR-423-5p—Cacna2d2—[Ca^2+^]i pathway plays an important role in mediating the effects of cocaine on BP and aortic stiffness.

## 3. Discussion

The CV consequences of cocaine exposure have been well documented (recently reviewed in [51]). These include both short term (acute) effects, including acute chest pain, acute coronary syndrome (ACS), hemorrhagic and ischemic stroke, and cardiac arrhythmias [52], as well as long-term consequences, including HTN, aortic stiffness, increased left ventricular mass, and bradycardia [9,10,11]. Cocaine acts through multiple mechanisms to exert it’s effects on the CV system [53,54]. Cocaine stimulates the sympathetic nervous system by increasing the sensitivity of adrenergic nerve terminals to norepinephrine and inhibiting catecholamine reuptake at sympathetic nerve endings [12]. These sympathomimetic effects led to the stimulation of cardiomyocyte adrenergic receptors, resulting in increased heart rate, BP, and vasoconstriction, which, in turn, increases myocardial oxygen demand. The sympathomimetic effects can cause elevated levels of the vasoconstrictor protein endothelin-1 [55], increased ROS levels [33], inhibited nitric oxide (NO) synthase [21], impaired acetylcholine-induced vasorelaxation [56], and dysregulated intracellular calcium levels [57]. Although the sympathomimetic mechanisms contribute to CV disorders, they cannot explain the full extent and the diversity of cocaine-induced CV phenotypes.

Accumulating experimental and clinical evidence supports a key role for miRNAs in regulating a wide variety of cellular processes, including the response to cocaine exposure [58,59,60,61]. For example, miR-212 was found to be elevated in the dorsal striatum following cocaine exposure regulating compulsive-like cocaine self-administration [34]. This effect was mediated through the modulation of cAMP response element binding protein (CREB) signaling. MiR-495 was shown to be down regulated in response to an acute exposure to cocaine leading to an upregulation of the miR-495 target genes—brain-derived neurotropic factor (BDNF), Calcium/Calmodulin Dependent Protein Kinase II Alpha (CaMKIIα), and Activity Regulated Cytoskeleton Associated Protein (Arc) [62]. The overexpression of miR-124 or let-7 in the nucleus accumbens attenuated cocaine-induced conditioned place preference in rats [63]. These cocaine-responsive miRNA regulatory pathways were all studied in the central nervous system. Far less is known about the role of miRNAs in mediating the cocaine effects in the CV system. Recently, we demonstrated that miR-30c-5p was upregulated in the aortas of cocaine exposed mice leading to the downregulation of Me1 and an increase in ROS [33]. This elevation of ROS contributed, in part, to the increased BP and aortic stiffness seen in cocaine- or CM-exposed mice. This cocaine-induced elevation in BP and aortic stiffness could be partially abrogated by pretreating the cocaine-exposed mice with a miR-30c-5p antagonist. In an effort to uncover additional miRNAs that could mediate the cocaine effects in the CV system, we focused on the analysis of miR-423-5p, a miRNA that was implicated in heart failure, coronary artery disease, pericardial effusion after cardiac surgery [64], as well as ischemia/reperfusion and the induction of apoptosis [65]. We found that miR-423-5p was markedly decreased in aortas from mice treated with a 10-day course of cocaine. Furthermore, miR-423-5p expression was anti-correlated with the calcium voltage-gated channel component Cacna2d2, a predicted target of miR-423-5p. Luciferase reporter assays showed that Cacna2d2 was a direct target of miR-423-5p. By modulating Cacna2d2 expression, miR-423-5p was found to control [Ca^2+^]i and, in turn, SMC contractility. It is well established that the regulation of [Ca^2+^]i levels plays a critical role in regulating SMC contractility and the development of myocardial ischemia, infarction, HTN, and arrhythmia [20,48,57,66]. L-type Cav1.2 channels (LTCCs) are the principal channels involved in mediating the influx of Ca^2+^ and the regulation of myogenic tone [45,46,47]_._ Overexpression of miR-423-5p resulted in reduced SMC contractility in the presence of cocaine. Conversely, the silencing of miR-423-5p expression resulted in increased SMC contraction. Similar to miR-423-5p overexpression, Cacna2d2 silencing using small interfering RNA resulted in decreased SMC contraction. Importantly, the overexpression of miR-423-5p from a SMC specific promoter (*Sm22*α) was able to partially abrogate the elevation of systolic and diastolic BP and aortic stiffness observed in a mouse model of cocaine use/abuse. These results showed that the miR-423-5p—Cacna2d2—intracellular calcium concentration ([Ca^2+^]i) pathway serves as an important regulator of BP and aortic stiffness in response to cocaine.

Voltage-gated calcium channels (VGCCs) are protein complexes composed of a main, pore-forming α1 subunit and auxiliary α2δ and β subunits with these auxiliary subunits thought to modulate the biophysical properties of the channel and participate in the trafficking and surface expression of the calcium channel [67]. Previous studies showed that Cacna2d2 was a direct target of another miRNA, miR-1231 [68]. Zhang and colleagues (2017) showed that miR-1231 was overexpressed in both human hearts following MI and in the hearts of rats subjected to an experimental model of MI [68]. Suppression of miR-1231 expression in rat hearts abrogated arrhythmias in the MI model by modulating Cacna2d2 levels. These results indicate that Cacna2d2 may serve as the intersection point between several miRNA pathways in CV disease facilitating the modulation of this key Ca^2+^ channel component in response to different environmental challenges. The effects of modulating Cacna2d2 activity are not restricted to CV tissue. In the nervous system, enhanced expression of the α2δ-2 subunit increased CaV2 channel density at the presynaptic active zone, leading to increased cytosolic- free calcium concentrations [69]. The ducky (du) mutation of Cacna2d2 in mice showed a severe phenotype characterized by cerebellar ataxia, epilepsy, reduced body weight, and premature death [70]. Cerebellar Purkinje cells of du mice had 35% smaller whole-cell Ca^2+^ currents mediated by P-type (Cav2.1) VGCC calcium channels and abnormal morphology of their dendritic trees [71]. Furthermore, the α2δ-2 subunit regulates the Ca^2+^ current amplitude and impacts the gating of L-type (Cav1.3) VGCC calcium channels in du mice [72]. Cacna2d2 null mice showed growth inhibition, cerebellar degeneration, elevated susceptibility to seizures, reduced life span, and cardiac abnormalities, including alterations in heart rate (bradycardia).

One of the limitations of this study was that miRNAs, including miR-423-5p, have the potential to silence multiple target genes. This could confound the phenotypic effect of the modulation of a specific miRNA. For example, Zhang et al. (2021) [73] showed that miR-423-5p levels were upregulated in exosomes from the plasma of bicuspid aorta valve (BAV) patients and could regulate TGF-β signaling by decreasing SMAD2 expression. Furthermore, miR-423-5p was shown to regulate cell apoptosis by enhancing caspase 3/7 activity and impairing mitochondrial functionality by directly targeting Myb-related protein B (MYBL2) expression in a model of hypoxia/reoxygenation [74]. MicroRNA-423-5p was also shown to target O-GlcNAc transferase to induce apoptosis in cardiomyocytes in response to oxidative stress (H_2_O_2_ treatment) [64]. These results showed that miR-423-5p plays multiple roles in CV phenotypes in response to different environmental cues. This also raises the possibility that miR-423-5p is targeting additional mRNAs that may contribute to the overall functionality of the SMCs. It is clear from the results that we presented that the miR-423-5p—Cacna2d2 pathway acts to modulate SMC contractility in the context of cocaine. However, we cannot eliminate the possibility that miR-423-5p may act through additional targets to modulate SMC function that may influence contractility. In addition, cocaine exposure can influence multiple miRNAs in addition to miR-423-5p. Cocaine exposure has multifactorial effects on the CV system by engaging multiple miRNA-mRNA pathways that target different aspects of CV cell health and functionality. We already uncovered important roles for the miR-30c-5p—Me1—ROS pathway and, in this work, the miR-423-5p—Cacna2d2—[Ca^2+^]i in the CV response to cocaine exposure. Another limitation of this study was the applicability to humans. Although this study showed the relationship between the miR-423-5p—Cacna2d2 pathway and SMC contractility, as well as BP and aortic stiffness in mice, it remains to be seen if this same pathway regulates SMC functionality in humans. Future studies analyzing the transcriptome of cardiomyocytes from humans that were exposed to cocaine will help to determine if this pathway plays a role in the human disease. Recent advances in the therapeutic application of RNAs raised the possibility of harnessing the regulatory potential of miRNAs for the treatment of disease (reviewed in [75]). The therapeutic modulation of the miR-423-5p—Cacna2d2 and miR-30c-5p—Me1 pathways may prove to be beneficial in the treatment of cocaine-induced CV phenotypes, including HTN and vascular senescence (aortic stiffness).

## 4. Materials and Methods

### 4.1. Animals

Male C57BL/6 mice aged 8–10 weeks were purchased from Charles River Laboratory (Hollister, CA, USA). Animals were treated according to National Institute of Health guidelines. Mouse protocols were approved by the Animal Care and Use Committee (IACUC) of the University of Miami Miller School of Medicine. Mice received intraperitoneal (I.P.) injections of cocaine, CM (20 mg/kg, NIDA Drug Supplied), or saline for 10 consecutive days. After the last injection, mice were measured for PWV and euthanized. Aortas were collected. In parallel animal experiments, mice received tail vein injection of lentiviruses encoding miR-423-5p or empty vector control (both from Biosettia, San Diego, CA, USA) driven by SMC-specific promotor *SM22*α at the dose of 3 × 10^5^ IU per mouse daily, every other day over the period of 5 days for a total of 3 injections. Then, mice received daily injection of cocaine or saline for 10 consecutive days as described above.

### 4.2. Blood Pressure and Pulse Wave Velocity Measurement

BP and PWV were measured as previously described [34]. Briefly, CODA BP monitor (Kent Scientific, Torrington, CT, USA) was used to measure mouse systolic and diastolic BP followed the manufacturer’s instructions. BP was measured before the first injection of cocaine (baseline BP), and then, at days 1, 3, 5, 7, and 10 one hour after the cocaine injection. PWV was measured before the first injection and at the end of the last injection. Mice were anesthetized with 2% isoflurane and laid on a platform. Blood flow velocity was measured at the middle level of the ascending aorta. By placing a 420–440 MHZ Doppler probe to the right of the upper sternum, the aortic arch velocity signal was obtained. Three measurements were recorded for each mouse.

### 4.3. Lentivirus Production and SMC Cell Lines

C57BL/6 mouse primary aortic SMCs were purchased from Cell Biologics (Cell Biologics, Chicago, IL, USA). Cells were cultured to passage 4 in the Complete Smooth Muscle Cell Medium (Cell Biologics), in the culture plates coated with gelatin (Cell biologics). The lentiviral vectors for the scrambled miR-Ctrl, miR-423-5p, miRZip-Ctrl, and miRZip423-5p (anti-miR-423-5p) were purchased from System Biosciences Inc. (Palo Alto, CA, USA). Lentiviral vectors encoding miR-Ctrl, miR-423-5p, miRZip-Ctrl, miRZip-423-5p were generated by co-transfection of the specific lentiviral vector with the lentiviral packaging plasmid, pCMV-D8.2, and the vesicular stomatitis virus envelope glycoprotein (VSVg) expression construct, pCMV-VSV-G, at a ratio of 3:2:1 in HEK 293T cells (Millipore Sigma, Burlington, MA, USA) using Lipofectamine 2000 (Thermo Fisher Scientific, Waltham, MA, USA). Then, 48 h after transfection, culture medium was collected, filtered, and concentrated using the Lenti-X™ Concentrator (Takara Bio, San Jose, CA, USA). Viral particles were suspended in fresh DMEM medium and stored at −80 °C. ShRNA for Cacna2d2 and the control shRNA lentiviral particles were purchased from Santa Cruz Biotechnology. To establish stable SMC cell lines, SMCs were transduced with specific lentiviral vectors. Briefly, SMCs were seeded at the density of 2 × 10^5^ per well in 6-well plates. After 2 h, viral vectors were added to the cell culture medium at an MOI of 2 together with 5 μg/mL polybrene (Millipore Sigma). The stably transduced cells were selected by treating the cells with 10 μg/mL puromycin.

### 4.4. Site-Directed Mutagenesis in Cacna2d2 and Luciferase Reporter Assays

Full length wild Cacna2d2-3′UTR was cloned downstream of a Gaussia luciferase reporter gene in the pEZX-MT05 vector (GeneCopoeia, Baltimore, MA, USA). Site-directed mutagenesis was used to introduce mutations into the putative miR-423-5p binding sites on the Cacna2d2 3′UTR using the QuickChange II XL site-direct mutagenesis kit (Agilent, Santa Clara, CA, USA) according to the manufacturer’s protocol. The pEZX-MT05 vector contains a constitutively expressed secreted alkaline phosphatase (SeAP) reporter gene, which served as an internal control for transfection normalization. Luciferase reporter assays were performed by co-transfection of miR-423-5p or miR-Ctrl construct (System Biosciences, Palo Alto, CA, USA) with either the wild-type Cacna2d2-3′UTR or the Cacna2d2 3′UTR bearing mutations in the miR-423-5p binding sites (Cacna2d2 mut 3′UTR) in HEK 293 cells (Millipore Sigma). Briefly, HEK 293 cells were seeded at 32,000 cells/well in 96-well plates and cultured overnight. The following day, the miR-423-5p or miR-Ctrl vector was cotransfected with either the Cacna2d2-3′UTR or the Cacna2d2 mut 3′UTR reporter vectors using Lipofectamine 2000 (Thermo Fisher Scientific) according to the manufacturer’s protocol. Luciferase and alkaline phosphatase activities were assayed using the Secrete-Pair^TM^ Dual Luminescence assay kit (Genecopoeia) and read on the Centro XS3 LB960 Microplate Luminometer (Berthold Technologies, Bad Wildbad, Germany).

### 4.5. Real-Time Quantitative PCR (RT-PCR) for mRNAs and miRNAs

Total RNA containing miRNAs was extracted from untransfected SMCs and cells transduced with miR-423-5p, miRZip-423-5p, scramble miR-Ctrl, miRZip-Ctrl, shRNA-Ctrl, shRNA-Cacna2d2 using the miRNeasy Mini Kit (Qiagen, Germantown, MD, USA) according to the manufacturer’s instructions. RNA concentrations were assessed using the NanoDrop™ 2000 Spectrophotometer (Thermo Fisher Scientific). Complementary DNA was produced using the High-Capacity cDNA Reverse Transcription kit (ABI) according to the manufacturer’s protocol. Quantitative real-time PCR (qRT-PCR) analysis was performed using the IQ SYBR Green Supermix (Bio-Rad, Hercules, CA, USA) according to the manufacturer’s protocol and read on the IQ5 multicolor Real-Time PCR Detection system (Bio-Rad). The Primers for Cacna2d2 were: Cacna2d2 forward primer: 5′-ccgctcttgctcttgctg-3′; Cacna2d2 reverse primer: 5′-ccagtgctgcatcgtgtg-3′. GAPDH was used as an internal control. Quantitation of miR-423-5p expression levels was assessed using the Taqman™ MicroRNA Reverse Transcription kit (Thermo Fisher Scientific) and the specific Taqman™ MicroRNA Assay kit and the Universal PCR Master Mix No AmpErase UNG (both Thermo Fisher Scientific). The RT-PCR reaction were performed on ABI 7900HT Fast Real-Time PCR system. The U6 small nucleolar RNA was used as the small housekeeping RNA reference gene for normalization of sample input [76,77,78].

### 4.6. Cytosolic Free Calcium Measurement

The intracellular free Ca^2+^ fluorescence images were obtained by free Ca^2+^ sensitive Fluo3-AM green fluorescence staining and the intracellular free Ca^2+^ concentrations were measured by FACS. The Fluo-3AM (Thermo Fisher Scientific) stock solution was prepared by first dissolving 50 μg of Fluo-3-AM dye in 35 μL DMSO. An amount of 14 μL of 10% Pluronic F-127 (Thermo Fisher Scientific) was added to the dissolved Fluo-3-AM solution and was then mixed with 117 μL of PBS. Cells were cultured and treated with cocaine (150 μm) or CM (150 μm) for 48 h and were then washed and stained with Fluo3-AM solution for 40 min at 37 °C. Fluorescence images were obtained using the EVOS FL Auto Cell Imaging System (Thermo Fisher Scientific). For FACS analysis of intracellular free Ca^2+^, cells were cultured and treated in the same way as Fluo3-AM staining. Briefly, cells were collected and washed with 1 × HBSS containing 1% fetal bovine serum then suspended in 1.0 mL of 1 × HBSS containing 2.5 μg/mL of Fluo-3-AM dye and 2.5 μm of the anion carrier inhibitor probenecid (Thermo Fisher Scientific) and incubated for 45 min at room temperature on an orbital shaker in the dark, washed and resuspended in 1 × HBSS containing probenecid, and incubated for 20 min in the dark. Fluorescence intensity in the stained cells was measured by FACS analysis. Ionomycin (Millipore Sigma) was used as a positive control and was added to the suspended cells 10 min before imaging and FACS analysis.

### 4.7. Collagen Gel Contraction Assay

The ability of SMCs to contract in response to CM, cocaine, or the calcium channel blocker Nimodipine (NM) was evaluated by using CytoSelect^TM^ 24-Well Cell Contraction Assay Kit (Cell Biolabs, San Diego, CA, USA) according to the manufacturer’s protocol. Briefly, SMCs were harvested and suspended in cell culture medium at 1 × 10^6^ cells/mL. Collagen gel working solution was produced according to the manufacturer’s instructions. Two parts of suspension cells were mixed with eight parts of collagen gel working solution to make cell contraction matrix and 0.5 mL of the cell contraction matrix was added to each well of the 24-well cell contraction plate. Collagen gels were allowed to solidify at 37 °C in 5% CO_2_ for 1 h. After the collagen gel solidified, 1.0 mL of culture medium containing 150 µM of cocaine, 150 µM of CM, or ET-1 was added atop the collagen lattice. Following the 48 h treatment, the gel was gently released from the sides of the culture plate and the contractility of the matrix was measured by digital imaging. The contractibility of SMCs infected with lentiviruses containing miR-Ctrl, miR-423-5p, miRZip-423-5p, shCtrl, shCacna2d2 was evaluated and compared. Images were evaluated using ImageJ (NIH). All experiments were performed in triplicates.

### 4.8. Immunofluorescence Staining

Immunofluorescence staining was performed on the frozen aortic sections. Briefly, mice were sacrificed and the aortas were flash frozen. Slides were obtained by cutting blocks in CM1850 microtome (Leica Biosystems, Deer Park, IL, USA). Frozen sections were fixed in ice cold 4% paraformaldehyde for 10 min at 4 °C, permeabilized in 0.3% Triton X-100/PBS for 5 min and incubated in 5% BSA for 30 min at room temperature to facilitate the blocking of nonspecific binding. Subsequently, slides were incubated with primary Cacna2d2 antibody at 1:50 dilation (Biorbyt, Durham, NC, USA) or isotype control at 4 °C overnight. Slides were washed with PBS and incubated with goat anti-rabbit AlexaFluor 488 diluted 1:300 (Molecular Probes, Grand Island, NY, USA) and Hoechst 33,342 dilution of 1:300 (Molecular Probes, Grand Island, NY, USA) for 60 min, washed, and mounted with Permount™ mounting media (Fisher Scientific, Waltham, MA, USA). Images were acquired using the EVOS FL Auto Cell Imaging System.

### 4.9. Statistical Analysis

All results are expressed as mean ± standard deviation (SD). Student’s two tailed *t*-test was used for all statistical analysis. Statistical analysis was performed by using SPSS16.0 computer software. A *p*-value < 0.05 was considered as statistically significant.

## Data Availability

The data presented in this study are available upon request from the corresponding author.

## Supplementary Materials

The following supporting information can be downloaded at: https://www.mdpi.com/article/10.3390/ijms24076584/s1.
