# Supplementary material for: MicroRNA-423-5p Mediates Cocaine-Induced Smooth Muscle Cell Contraction by Targeting Cacna2d2
Source: Int J Mol Sci. 2023 Apr 1;24(7):6584. doi: 10.3390/ijms24076584 (PMC10094933; doi:10.3390/ijms24076584)
Supplement: Supplementary file 1 [file ijms-24-06584-s001.zip › ijms-2257262-supplementary.pdf]

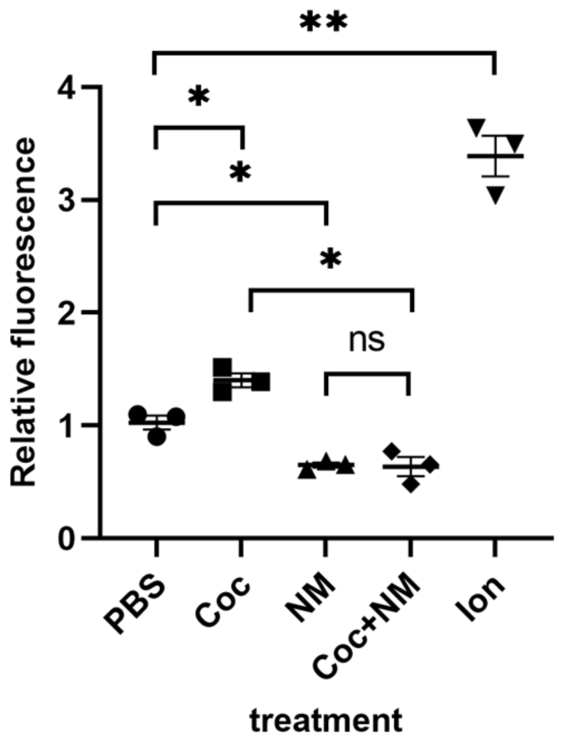


**Figure S1.** SMCs were treated with cocaine, NM, either individually or combined, FACS quantification of free $Ca^{2+}$-specific fluorescence emission shows that NM blocks the calcium channel and decreased intracellular calcium $[Ca^{2+}]_i$ compared with PBS. NM also abrogates the increasing effect caused by cocaine. Ionomycin treated cells were used as positive control. (ns: not significant; *$p<0.05$, **$p<0.01$).
